# A review of our journal in 2026

**DOI:** 10.4102/ajod.v15i0.2249

**Published:** 2026-10-31

**Authors:** Aimee V. Stewart

**Affiliations:** 1 Department of Physiotherapy, School of Therapeutic Sciences, Faculty of Health Sciences, University of the Witwatersrand, Johannesburg, South Africa

This year has been another good year for *African Journal of Disability*. We are increasingly having articles submitted from other African countries as well as from Southeast Asia. This is such valuable growth for the journal, given how many of our articles illustrate the difficulties faced by people with disabilities in middle- to low- income countries.

We are delighted that we have continuous submissions to the journal from such a wide variety of researchers in the disability field. Our authors range from educators to therapists to policymakers, amongst others. This is important so that we always illustrate the wide range of disability issues across both the lifespan and economic landscape. The disability issues cover both physical and intellectual disability and range in studies from children to adults.

Most of our articles are either original research or scientific literature reviews. This is such a healthy development as it shows that research is continuously being done in this field and is growing and developing in many of our institutions here and elsewhere.

Our articles range from reading difficulties of children with dyslexia to a consideration of how skills training has the added benefit of increasing personal empowerment and independence, thus improving the quality of life of people with disabilities. Return to work after stroke has previously been considered, but the article in our journal considers the difficulties of return-to-work post-stroke in a low-income country. This touches on the importance of employment opportunities for all. In addition to employment, one of the most self-empowering aspects for all of us is to achieve in areas other than employment, such as in sports. Sports for people with disabilities are growing worldwide, and we welcomed some articles discussing sport in this important area.

Given that this journal is published on the African continent, many of our submissions deal with the problems of poverty and disability, including the difficulty of accessing required support in both poor urban and rural communities. The lack of appropriate support for people with disabilities is an ongoing challenge and one which all researchers and workers in the field grapple with daily.

We also have articles on disability policy issues and on considering disability from a human rights perspective. The wide view of disability is so important, and we encourage our authors from all areas of research to consider working and publishing in this important field.

Future studies now need to consider interventions to improve the lives of people with disabilities. We have had many articles over the years describing the status quo in different areas of difficulty but very few interventions. Now our researchers should consider interventions that are appropriate to start the improvement needed by so many and that include the voice and aspirations of people with disabilities themselves.

Our special edition this year is titled: *Disability, spirituality and belonging*. This has shown yet other important aspects to consider in this field of study. The edition was edited by Dr Chioma Ohajunwa, Nafisa Mayat and Adele Ebrahim.

As always, the editorial team needs to thank our reviewers, who are the backbone of any scientific journal. Scientific journals are wholly dependent on peer review to ensure the credibility and standing of the journal. We are so grateful to the reviewers who agree to review, as we could not function without them. We would urge all our reviewers to please respond positively when asked to review, as this ensures that the review process goes smoothly. We appreciate, however, that we are always asking busy people to review, and that you probably have many demands on your time.

The editorial team are thanked for their commitment, overseeing the review process as well as editing and making decisions about acceptance or refusal of an article. These academics are also always busy, and we are grateful for the time that they give so wholeheartedly to the journal.

The publisher, also needs a word of gratitude for their ongoing support and commitment to making our journal a good one. Finally, to our readers, we hope that you enjoy the published articles and find them useful and relevant to your work in the disability research field.

Best wishes for the year ahead as we continue to highlight the important, essential research needed in the disability field. We look forward to more submissions from our authors.

